# Gastric diverticulum of the antrum: An unusual endoscopic finding

**DOI:** 10.1002/ccr3.1910

**Published:** 2018-11-05

**Authors:** Faidon‐Marios Laskaratos, Hanan El‐Mileik

**Affiliations:** ^1^ Centre for Gastroenterology Royal Free London NHS Foundation Trust London UK; ^2^ Endoscopy Unit Chartwell Private Hospital Southend‐on‐Sea, Leigh‐on‐Sea UK; ^3^ Gastroenterology Department Queen’s Hospital, Barking Havering and Redbridge NHS Trust Romford UK

**Keywords:** antrum, endoscopy, gastric diverticulum, gastroscopy

## Abstract

Gastric diverticula are rare and may sometimes cause diagnostic confusion. Most cases are asymptomatic and diagnosed incidentally. However, sometimes they can cause a variety of clinical manifestations and may be complicated by bleeding, perforation, or malignancy. Therefore, clinicians should be aware of this unusual finding and the available management options.

A 26‐year‐old female with no significant past medical history presented with non‐specific abdominal discomfort, which had demonstrated a partial response to a trial of proton pump inhibitor (PPI) therapy. A gastroscopy was performed which showed a small (5‐6 mm in diameter) diverticulum in the antrum (Figure 1A,B).

**Figure 1:**
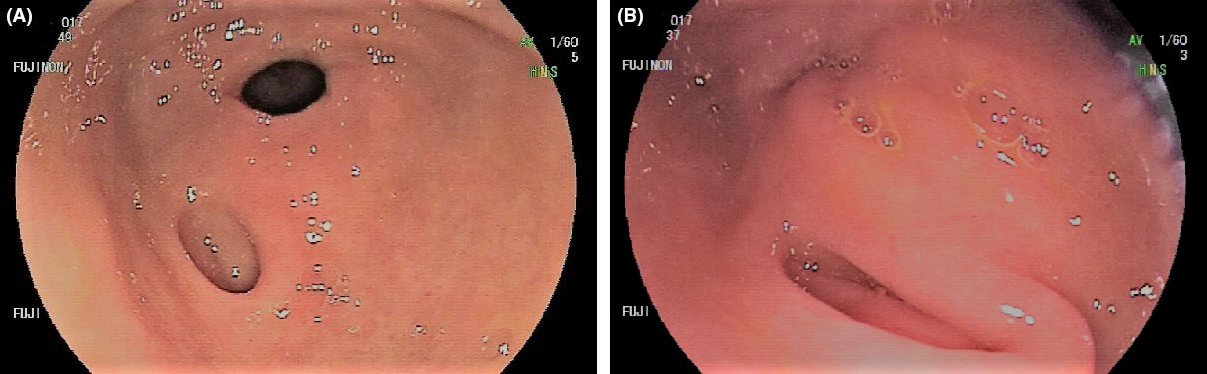
(A and B) Endoscopic view of the antrum demonstrating a small diverticulum adjacent to the pylorus

Gastric diverticula are very rare with a reported prevalence of 0.01%‐0.11% at upper gastrointestinal endoscopy and 0.03%‐0.3% at autopsy studies.^1^ The majority (75%) of true gastric diverticula are located in the fundus, while in extremely rare cases they have been reported in the antrum.^2^ False diverticula (pseudodiverticula) are less common and typically associated with other gastrointestinal disorders, such as peptic ulcer disease or malignancy.^1^ Most cases are asymptomatic, but symptomatic patients can present with a variety of clinical manifestations, such as abdominal pain, nausea, vomiting, dyspepsia, weight loss, bleeding, or even perforation. The mechanisms by which gastric diverticula can cause symptomatology are somewhat unclear but include development of complications and food retention with bacterial overgrowth (which can lead to dyspepsia, belching, and halitosis).

Management of symptomatic patients is usually conservative with PPI therapy, but in cases of gastric diverticula complicated by bleeding, perforation, or malignancy, surgery (open or laparoscopic resection) is recommended.

## CONFLICT OF INTEREST

None declared.

## AUTHOR CONTRIBUTION

Both authors contributed equally to the conception and preparation of the manuscript.
